# Relationship between floating toe score and performance in track and field athletes

**DOI:** 10.1371/journal.pone.0314087

**Published:** 2025-01-24

**Authors:** Yohei Yamazaki, Hiroaki Noro, Keiichiro Hata, Misato Ishikawa, Hiyo Inaba, Kazuhiko Yamazaki, Ryoichi Murayama, Hideyuki Arikawa, Aya Miyamoto, Toshio Yanagiya

**Affiliations:** 1 Graduate school of Health and Sports Science, Juntendo University, Chiba, Japan; 2 Institute of Health and Sports Science & Medicine, Juntendo University, Chiba, Japan; 3 Faculty of Health and Sports Science, Juntendo University, Chiba, Japan; 4 Faculty of Physical Education, International Budo University, Chiba, Japan; 5 Faculty of Education, Saitama University, Saitama, Japan; 6 Department of Physical Education, International Pacific University, Okayama, Japan; eCampus University: Universita degli Studi eCampus, ITALY

## Abstract

This study explored the relationship between floating toes and athletic performance among collegiate track and field athletes. A total of 422 athletes (sprinters, jumpers, and distance runners) and 136 controls participated in this study. Plantar surface images were captured using a specially designed foot scanner during standing to calculate the floating toe score. The score, which sums the floating toe points of all toes, categorizes them into ‘floating toe’, ‘incomplete contact’, or ‘normal toe’. The World Athletics score served as a measure of athletic performance. Overall, collegiate track and field athletes had a significantly higher mean floating toe score (14.22 ± 4.87 points) than controls (11.06 ± 6.05 points) (*p* < 0.05), suggesting that fewer floating toes may confer a performance advantage. Within the track and field events, sprinters and jumpers showed significantly higher floating toe scores than distance runners (*p* < 0.05), emphasizing the variations in floating toes across different events. Although no significant correlation between floating toe score and World Athletics score was observed overall, a significantly negative correlation was found among distance runners (r = -0.25, *p* = 0.006), indicating that distance runners with higher athletic performance tend to have more pronounced floating toes. These findings suggest a nuanced relationship between floating toes and various aspects of track and field performance, particularly in distance running.

## Introduction

‘Floating toe’ is defined as the condition in which the toes do not fully contact with the ground during static standing [1]. It is commonly assessed using the floating toe score, a 20-point scale that measures the number of toes in contact with the ground when standing. A score of 10 or less is considered a floating toe [1–5]. This condition has a high prevalence in Japan, with reports of 40.3% in children [6] and 27.7% in adults [1]. The difference in prevalence between children and adults may be related to the increase in lower limb muscle strength with age [6].

Many causes of floating toes have been reported. Physical factors [5, 7], lifestyle effects [8], and even surgical procedures such as Weil osteotomy [9, 10] have been associated with the condition. One key factor is excessive dorsiflexion at the metatarsophalangeal (MTP) joint, often resulting from inadequate plantarflexion force. This leads to a lack of toe support, resulting in decreased postural stability when standing [11] and reduced weight transfer when walking [12]. In addition, individuals with floating toes often have lower toe grip strength [6], which further worsens these stability issues.

Despite these findings, the effect of floating toes on athletic performance remains unclear. While previous studies have highlighted the potential influence of floating toes on motor function, particularly in tasks requiring foot stability [12, 13], the relationship between floating toes and athletic performance has not been thoroughly investigated. This is particularly concerning for athletes, where any instability in foot mechanics could directly impact performance. If highly competitive athletes have fewer floating toes, then it is likely that toe contact with the ground plays an important role in athletic function.

The purpose of this study was to examine the relationship between floating toes and athletic performance, specifically in running events, in track and field athletes. We hypothesized that floating toes are detrimental to runners, particularly in events where foot stability and force generation are critical. The impact of floating toes on athletic performance is not fully understood. This study aims to clarify whether preventive interventions may be necessary to improve performance in running events. The results of this study will also help determine whether early intervention for floating toes is necessary to improve future motor function in children, especially if this condition does not significantly impair athletic performance.

## Methods

### Participants

Sample size estimation was conducted using G*Power software (version 3.1.9). By setting the level of significance to 0.05, the statistical power to 0.80, and the medium effect size to 0.30, the estimated required sample size was calculated to be 84.

This study recruited 558 participants: 422 collegiate track and field athletes who regularly do the specialised training and 136 students majoring in physical education and sports science with exercise habits as a control group (CON). The track and field athletes were further divided into three event categories: 194 sprinters (including 100m, 200m, and 400m specialists), 107 jumpers (long jump, triple jump, and high jump), and 121 distance runners (800m, 1500m, 5000m, and 10000m). These categories were based on the athletes’ primary events to ensure that each group’s specific demands were appropriately considered in the analysis. These athletes include 13 world level competitors in their categories. These participants were recruited from several different universities to minimize the potential bias that could arise from environmental, institutional, or training factors. By collecting data from athletes across multiple universities, we aimed to reduce the influence of specific training conditions, competitive levels, and daily training routines that may vary significantly between institutions. This approach helps to ensure that the results reflect broader trends rather than being influenced by the unique characteristics of a single university’s program. The official personal best record (PB) was self-reported and verified online. Afterward, their obtained PB was converted to World Athletics (WA) score [14, 15] to compare the performance between the different track and field events. The characteristics of the participants and WA score by event are shown in Table 1.

**Table 1:** Physical characteristics and World Athletics (WA) score of participants (mean ± SD).

Group	n	Age	Height	Body mass	WA score
	(Male, female)	[years]	[cm]	[kg]	[points]
All track and field athletes	422 (361, 61)	20.7**…**±**…**1.5	172.6**…**±**…**7.3	63.9**…**±**…**7.9	921.5**…**±**…**113.9
Sprinters	194 (170, 24)	20.6**…**±**…**1.3	174.2**…**±**…**7.3	67.3**…**±**…**7.4	947.1**…**±**…**103.7
Jumpers	107 (74, 33)	20.7**…**±**…**1.5	171.1**…**±**…**7.8	63.8**…**±**…**7.5	912.9**…**±**…**101.9
Distance runners	121 (117, 4)	20.6**…**±**…**1.1	171.0**…**±**…**6.0	57.7**…**±**…**5.0	902.0**…**±**…**141.6
CON	136 (111, 25)	20.7**…**±**…**1.5	168.9**…**±**…**7.0	65.2**…**±**…**8.5	

This study was carefully designed and conducted in accordance with the ethical guidelines for research involving human subjects established by the Faculty of Health and Sports Science, Juntendo University, and adhering to the tenets of the Helsinki Declaration of 1964 and its subsequent amendments. Prior to participation, all potential participants received a detailed written explanation of the purpose of the study, procedures, and their rights, including the right to withdraw from the study at any time without consequence. Written informed consent was then obtained from each participant. The confidentiality and privacy of all participants’ data were rigorously protected; all personal identifiers were removed to ensure anonymity, and data were securely stored. Ethical approval for this research was granted by the Ethics Committee with approval code JU_SPO_Eth_No.27-20. The data was collected from September 24, 2015, through January 31, 2024.

### Data collection

As described by Yanagiya et al. [2], participants’ standing plantar surface images were captured using a commercially available specially made foot scanner (FootLook, FootLook, Japan; 1755px×2550px, 150dpi). Prior to imaging, participants were asked to clean the soles of their feet with alcohol wipes to ensure cleanliness and improve image quality. For imaging, participants stood on the scanner with their feet shoulder-width apart and maintained a static posture. They were instructed to hold their arms out to their sides and to focus their gaze on a marker placed at eye level on the wall approximately 2-m in front of them to consistent and standardized foot posture. The image acquisition process took approximately 10 s, during which time participants were required to maintain this specified posture to ensure consistency. The acquired plantar surface images were immediately stored on a secure computer system to ensure data integrity and privacy.

### Data analysis

**Fig 1:**
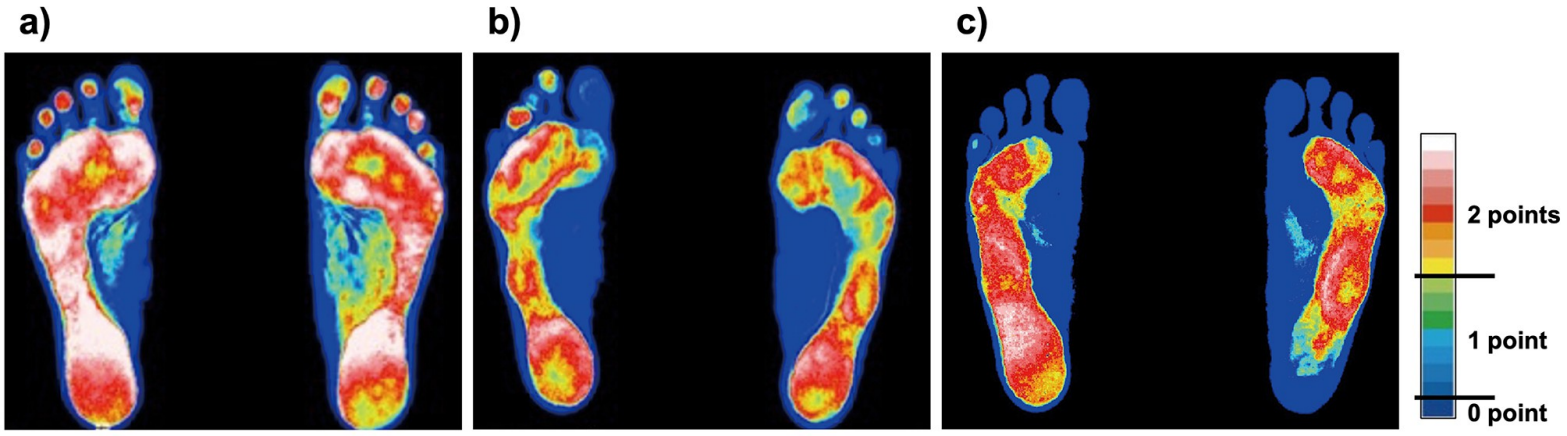
The examples of plantar pressure distribution indicate a) normal toe, b) incomplete contact, and c) floating toe. The images illustrate varying pressure levels across the plantar surface, with a color scale ranging from dark blue (no pressure) to white (highest pressure). This color scale is integral to evaluating floating toes, where specific colors correspond to different scores: yellow, red, or white indicate 2 points, green or light blue 1 point, and dark blue 0 point. This visualization aids in quantifying the severity and presence of floating toes based on observed pressure distribution.

The plantar surface images captured by the scanner were analysed using specialised software (FootLook2, Japan). The software estimates pressure distribution by analysing changes in color of the human sole skin under different pressure intensities on the foot, which is then visualized on a 16-level color scale. Each toe area was scored according to the criteria of Yanagiya et al. [2]: toes showing yellow, red, or white were scored 2 points, green or light blue was 1 point, and dark blue was no points (see Fig 1). The floating toe score was calculated as the sum of the floating toe points for each foot and both feet combined, with a maximum score of 20 points. A floating toe score equal to or above 18 points were classified as ’normal toe’; the scores between 11 and 17 as ’incomplete contact’; and 10 or below as ’floating toe’.

To ensure the accuracy and reliability of the score, intra- and inter-rater reliability was evaluated using intraclass correlation coefficients (ICCs) from a two-way random effects model. Intra-rater reliability was assessed by analyzing data five times for each of the 10 participants, and inter-rater reliability was assessed by two raters independently assessing the same 10 participants using identical criteria. The ICC values were exceptionally high at 0.93 for intra-rater and 0.91 for inter-rater reliability, indicating excellent agreement and consistency in the measurement process.

### Statistical analysis

Statistical analyses were performed using JASP software (JASP Team, 2023). Descriptive statistics for discrete variables were expressed as mean ± standard deviation (SD). The normality of the distribution of the floating toe score and the WA score was assessed using the Kolmogorov-Smirnov test, which indicated that neither variable followed a normal distribution. Therefore, due to the lack of normality, non-parametric tests were used for further analyses. The Kruskal-Wallis test was used to compare mean differences in floating toe score and WA score between different groups of participants. Where significant differences were found, post-hoc pairwise comparisons were made using the Mann-Whitney U test to determine specific group differences. In addition, the relationship between floating toe score and WA score in track and field athletes was examined using Spearman’s rank correlation coefficient. The statistically significant level was set at α = 0.05.

## Results

The overall mean floating toe score for track and field athletes was 14.22 ± 4.87 points, which was significantly higher than the control group’s mean of 11.06 ± 6.05 points (*p* < 0.001, See Table 2). Among the track and field events, sprinters had a mean floating toe score of 14.73 ± 4.49 points, jumpers 14.59 ± 5.06 points, and distance runners 13.08 ± 5.11 points. Comparisons within these groups showed that both sprinters and jumpers had significantly higher values than distance runners (*p* = 0.006 for sprinters vs. distance runners; *p* = 0.02 for jumpers vs. distance runners). All track and field event groups had significantly higher floating toe score than the control group (*p* < 0.05 for each comparison).

**Table 2:** Floating toe score across different groups: Track and field athletes (sprinters, jumpers, distance runners) and control group (mean ± SD).

Group	Floating toe score…[points]	
All track and field athletes	14.22**…**±**…**4.87	*
Sprinters	14.73**…**±**…**4.49	*, †
Jumpers	14.59**…**±**…**5.06	*, †
Distance runners	13.08**…**±**…**5.11	*
CON	11.06**…**±**…**6.05	

*: Significant difference from CON,

^†^: Significant difference from distance runners (*p* < 0.05)

**Fig 2:**
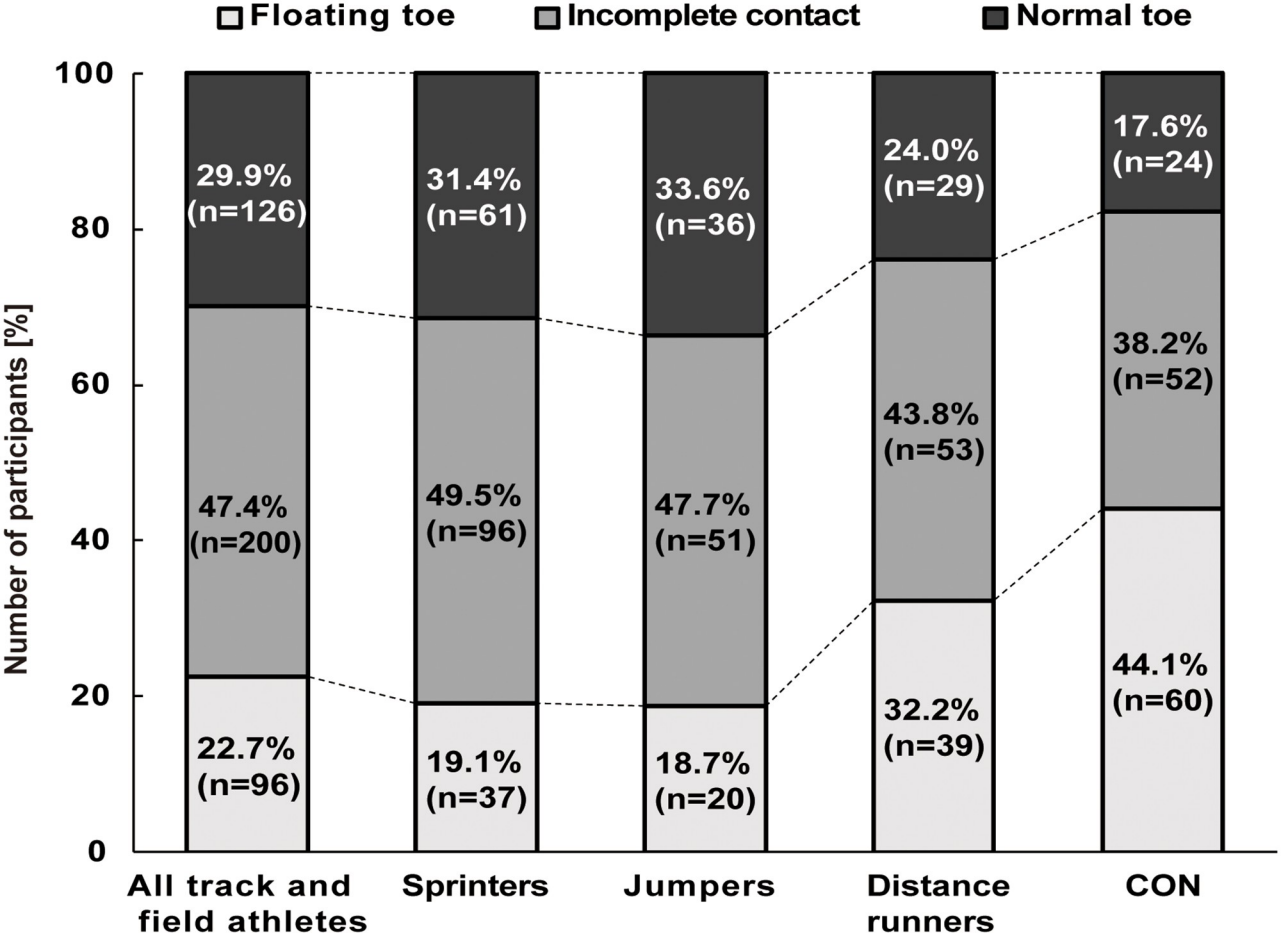
Distribution of participants by floating toe score categories across different groups: Track and field athletes (sprinters, jumpers, distance runners) and control group, represented in a bar chart.

The distribution of floating toe score categories—floating toe, incomplete contact, and normal toe—among the participant groups is shown in Fig 2. Of 422 athletes, the proportions of floating toe, incomplete contact, and normal toe were 22.7%, 47.4%, and 29.9%, respectively. In contrast, the control group (CON; n = 136) showed a higher proportion of floating toe at 44.1% followed by incomplete contact at 38.2% and normal toe at 17.6%.

Detailed analysis of the track and field events showed different proportions: Sprinters had 19.1% floating toe, 49.5% incomplete contact, and 31.4% normal toe. Jumpers had a similar distribution with 18.7% floating toe, 47.7% incomplete contact, and 33.6% normal toe. Distance runners had 32.2% floating toe, 43.8% incomplete contact, and 24.0% normal toe.

Notably, sprinters and jumpers had a higher proportion of normal toe compared to floating toe, whereas distance runners showed the opposite trend, with floating toe being more common. This pattern is clearly in contrast to the CON where the floating toe was most common.

**Fig 3:**
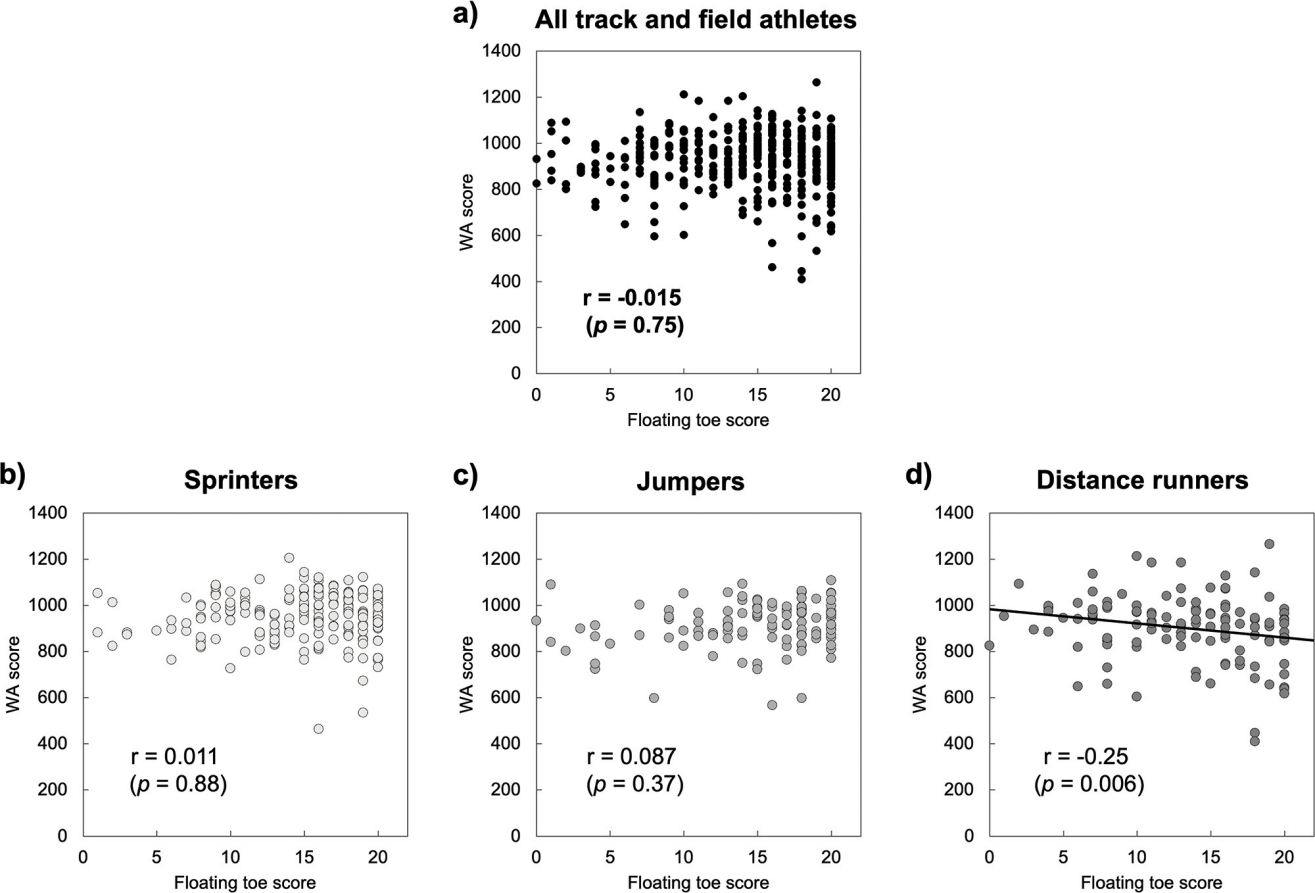
Relationships between World Athletics (WA) score and floating toe score analysed separately for different groups of track and field athletes. (a) All track and field athletes combined, (b) sprinters, (c) jumpers, and (d) distance runners.

The relationships between WA score and floating toe score were assessed and are shown in Fig 3. In the total group of 422 athletes, no significant correlation was found between WA score and floating toe score (r = -0.015, n = 422, *p* = 0.75; Fig 3a). This suggests that within this broad group, the WA score does not predict floating toe score. However, the results analysed by specific event categories showed a notable difference in distance runners. A significant negative correlation between WA score and floating toe score was found in this subgroup (r = -0.25, n = 121, *p* = 0.006; Fig 3d), indicating that higher WA score is associated with lower floating toe score in distance runners.

## Discussion

This study investigated the relationship between floating toes and athletic performance among collegiate track and field athletes. The main findings revealed that the average floating toe score among these athletes was 14.22 ± 4.87 points, indicating incomplete toe contact. Interestingly, sprinters and jumpers exhibited higher floating toe scores compared to distance runners, suggesting a potential link between toe contactf and specific athletic disciplines (Table 2). Moreover, when examining the correlation between floating toe score and WA score, no signifunicant correlation was found across the entire group of track and field athletes, nor within sprinters and jumpers (Fig 3a–3c). However, a significant negative correlation was observed in distance runners (Fig 3d).

Previous studies have suggested that floating toes in children may be caused by inadequate plantarflexion force at the MTP joint, and that this condition might resolve naturally as muscle strength increases with age [6]. However, our data challenge this assumption. Despite the high athletic ability and presumably greater muscle strength of the collegiate track and field athletes in our study, many still exhibited floating toes (Fig 2). This finding suggests that increased muscle strength alone may be insufficient to correct floating toes, contradicting the assumptions made in previous research. It indicates that other factors, such as biomechanics, foot structure, or specific athletic demands, may play a more significant role in the persistence of floating toes, even in high-performing athletes.

Previous studies have shown that floating toes can negatively impact motor functions such as posture maintenance, toe grip strength, and walking ability [6, 12, 13]. Previous research focusing on children, however, did not observe these effects [4], highlighting possible age-related differences. In contrast to these findings, our study suggests that incomplete toe contact may not significantly impair athletic performance in certain disciplines, such as sprinting and jumping (Fig 3b and 3c). This challenges the traditional view that floating toes universally impair motor function. However, the negative correlation observed between floating toe score and performance in distance runners (Fig 3d), suggests that a lower floating toe score may be associated with better performance in distance running. This may be due to a reduced need for force generation at the toes and greater energy efficiency.

Regarding the negative correlation between floating toe score and WA score in distance runners (Fig 3d), I hypothesize that while a high floating toe score, indicating better support from the toes, may be advantageous for maintaining posture during static standing, it may be unnecessary force during running. This increased force could hinder smooth weight transfer and forward motion. In contrast, a less stable posture during standing might allow distance runners to advance with less effort, leading to reduced energy consumption during running [16]. In sprinting, explosive force generation is crucial, and previous studies have shown that negative force exerted by the MTP joint contributes to subsequent propulsion [17]. However, in distance running, the emphasis is on minimizing energy expenditure to maintain speed over extended periods, which could explain the observed results.

There are several limitations to this study that must be acknowledged. Firstly, the cross-sectional nature of the study prevents us from establishing a causal relationship between floating toes and athletic performance. Longitudinal studies are needed to determine whether changes in floating toe conditions directly impact performance over time. Secondly, this study evaluated floating toes in a static standing position, and it remains unclear whether this condition persists during dynamic activities like running. Future research should assess floating toes in motion to better understand their impact on athletic performance. Additionally, this study did not analyze gender differences in floating toe scores due to the lack of sufficient data at this stage. Considering that females often have foot deformities like hallux valgus and potentially lower foot muscle mass [18], it is important to acknowledge that our current findings may not fully capture the influence of gender on floating toes and athletic performance. Therefore, we recommend that future studies further explore this aspect with a more comprehensive dataset to better understand the potential gender-specific effects. Lastly, the control group in this study consisted of students majoring in sports science, who likely have higher athletic abilities than the general population. Including a more diverse control group in future studies will help generalize the findings more effectively.

## Conclusion

This study provides new insights into the relationship between floating toes and athletic performance in collegiate track and field athletes. While the presence of floating toes did not universally impair performance, the specific impact varied across different athletic disciplines. These findings suggest that the role of floating toes in athletic performance may be more complex than previously thought. Further research is needed to clarify this relationship, focusing on dynamic assessments and potential gender differences.

## Supporting information

S1 Dataset (XLSX)
